# Alterations in Urine Metabolomics Following Sport-Related Concussion: A ^1^H NMR-Based Analysis

**DOI:** 10.3389/fneur.2021.645829

**Published:** 2021-08-19

**Authors:** Zachary R. Wanner, Cormac G. Southam, Prachi Sanghavi, Naveenjyote S. Boora, Eric J. Paxman, Sean P. Dukelow, Brian W. Benson, Tony Montina, Gerlinde A. S. Metz, Chantel T. Debert

**Affiliations:** ^1^Department of Neuroscience, Canadian Centre for Behavioural Neuroscience, University of Lethbridge, Lethbridge, AB, Canada; ^2^Department of Clinical Neurosciences, Cumming School of Medicine, University of Calgary, Calgary, AB, Canada; ^3^Cumming School of Medicine, Hotchkiss Brain Institute, University of Calgary, Calgary, AB, Canada; ^4^Winsport Medicine Clinic, Calgary, AB, Canada; ^5^Department of Chemistry and Biochemistry, University of Lethbridge, Lethbridge, AB, Canada

**Keywords:** sports-related concussion, urine metabolomics, ^1^H NMR spectroscopy, diagnostic tool, biomarker

## Abstract

**Objective:** Millions of sport-related concussions (SRC) occur annually in North America, and current diagnosis of concussion is based largely on clinical evaluations. The objective of this study was to determine whether urinary metabolites are significantly altered post-SRC compared to pre-injury.

**Setting:** Outpatient sports medicine clinic.

**Participants:** Twenty-six male youth sport participants.

**Methods:** Urine was analyzed pre-injury and after SRC by ^1^H NMR spectroscopy. Data were analyzed using multivariate statistics, pairwise *t*-test, and metabolic pathway analysis. Variable importance analysis based on random variable combination (VIAVC) was applied to the entire data set and resulted in a panel of 18 features. Partial least square discriminant analysis was performed exploring the separation between pre-injury and post-SRC groups. Pathway topography analysis was completed to identify biological pathway involvement. Spearman correlations provide support for the relationships between symptom burden and length of return to play and quantifiable metabolic changes in the human urinary metabolome.

**Results:** Phenylalanine and 3-indoxysulfate were upregulated, while citrate, propylene glycol, 1-methylhistidine, 3-methylhistidine, anserine, and carnosine were downregulated following SRC. A receiver operator curve (ROC) tool constructed using the 18-feature classifier had an area under the curve (AUC) of 0.887. A pairwise *t*-test found an additional 19 altered features, 7 of which overlapped with the VIAVC analysis. Pathway topology analysis indicated that aminoacyl-tRNA biosynthesis and beta-alanine metabolism were the two pathways most significantly changed. There was a significant positive correlation between post-SRC 2-hydroxybutyrate and the length of return to play (ρ = 0.482, *p* = 0.02) as well as the number of symptoms and post-SRC lactose (ρ = 0.422, *p* = 0.036).

**Conclusion:** We found that ^1^H NMR metabolomic urinary analysis can identify a set of metabolites that can correctly classify SRC with an accuracy of 81.6%, suggesting potential for a more objective method of characterizing SRC. Correlations to both the number of symptoms and length of return to play indicated that 2-hydroxybutyrate and lactose may have potential applications as biomarkers for sport-related concussion.

## Introduction

In the United States, the Center for Disease Control and Prevention estimates that 1.6–3.8 million concussions occur in sports and recreational activities annually (1). Currently, there is no single “gold standard” assessment or diagnostic marker to objectively determine if a patient has suffered a sport-related concussion (SRC) and, if so, how long it will take to recover. As such, SRC remains a clinical diagnosis based on symptoms and signs, which can be subject to interpretation. There is no current fluid biomarker available that quantitatively diagnoses SRC or predicts recovery following SRC.

Previous fluid biomarker studies hold promise in contributing to the understanding of the neurometabolic response to SRC. These studies have found significant alterations in tau protein (2, 3), s100B protein (2–5), neuron-specific enolase (2, 4), AMPAR peptide (6), glial fibrillary acidic protein (7), prolactin levels (8), plasma soluble prion protein (7), saliva cortisol (9), and plasma metabolites (10), in an attempt to develop more accurate diagnostic and prognostic methods for SRC.

A new method rapidly advancing clinical biomarker studies is metabolomics, which refers to the systematic study of chemical metabolites found in tissues and biofluids as the result of various biochemical reactions (11, 12). Metabolomics techniques, such as ^1^H NMR spectroscopy, have measured the response of these metabolites in plasma to SRC (10), resistance exercise (13), neurological diseases (14–21), and other disease states in animal models and humans (22–24). NMR spectroscopy is a method that studies a fundamental property, known as spin, of the various nuclei in a molecule—in this case the ^1^H nucleus. This technique is sensitive to both the immediate chemical environment (reported as the chemical shift) and the number of hydrogen atoms present (the integral or area of a peak) in each unique chemical environment in a molecule, thus providing unique fingerprints that can be used to both identify and quantify metabolites. As well, urine metabolomics have shown to be altered in many other diseases of the abnormal brain pathology (20, 21). ^1^H NMR metabolomics is valuable because it preserves the integrity of the sample, is non-biased, and can be performed on several biofluids including blood, cerebrospinal fluid, and urine (12). Utilizing a non-biased approach in metabolomics is key, as the multivariate model determines classification by examining only the data variability across the samples, which is based on the spectral areas provided (bins), in an automated fashion. Furthermore, ^1^H NMR metabolomics analysis of urine may be particularly useful as it is easily attainable in athletes, is the body's primary vehicle for excretion of small molecules (25), and is therefore sensitive to changes in biochemical pathways due to disease or injury.

Metabolite changes detected and quantified via ^1^H NMR urine metabolomics may be utilized in future technology for both the diagnosis and monitoring of SRC. For example, Numares Health (Boston, MA, USA) has recently filed for FDA approval for the use of an NMR-based metabolomics technology, known as AXINON GFR_NMR_, in the clinical setting with the goal of making NMR-based metabolomics diagnostics accessible to patients. In addition, lower-cost and portable benchtop-based NMR technology at smaller magnetic fields is creating the possibility of NMR devices with accessibility in both the local clinical and sports medicine (field-side) settings (26). The combination of the development of benchtop NMR methods and approval for use of larger NMR instruments in the clinical setting will enable access to and commercialization of NMR-based metabolomics biomarkers.

Emerging evidence in fluid metabolomics research suggests that metabolite signatures can accurately reflect central nervous system inflammation and neuronal injury (10, 14–18, 20, 21). Daley et al. (10) looked at metabolite changes in blood plasma following SRC and found changes in glycerophospholipids, which are associated primarily with membrane structures in the brain and inflammation. They concluded that, in the case of blood plasma, the metabolites identified by the liquid-chromatography/mass spectrometry method provided better predictive power when compared to ^1^H NMR. However, applying ^1^H NMR to study metabolomic changes in human urine provides significantly more metabolite information when compared to blood (49 metabolites in blood compared to 209 in urine) (25). Hence, based on both the increased metabolite information available via ^1^H NMR and the ease of access (noninvasive) of the biofluid, the use of urine as a biofluid should be explored before drawing any conclusion about the best technology for finding new quantitative biomarkers for SRC. Using ^1^H NMR analysis, the present study investigated alterations in the urinary metabolome of male winter-sport athletes pre-injury in comparison to post-SRC. A secondary objective of the study was to compare post-SRC urine metabolite profiles to clinical outcome measure of symptom burden and length of recovery.

## Materials and Methods

### Subjects

This study recruited 423 Canadian national team athletes, national development athletes, and amateur ice hockey players, ages 12–16 years, who were participating in the WinSport Concussion Clinic, from August 2015 to 2016. Exclusion criteria include age >40 and <12 years, female, a previous history of chronic medical conditions (e.g., metabolic or nephritic disorders), and neurological conditions such as stroke, seizure, moderate to severe traumatic brain injury, and/or congenital intracranial abnormalities. This was a longitudinal cohort study nested within a larger study assessing novel imaging techniques and robotic assessment in athletes. The greater study recruited from a larger age range (12–40 years) compared to the study described. The study was approved by the University of Calgary Conjoint Health Research Ethics Board.

### Clinical Protocol

#### Pre-injury Assessments

Urine samples were collected in the morning hours between 7 and 9 a.m. prior to the first meal to provide a non-concussed assessment in athletes that were currently training during their athletic season. Athletes were asked to only drink water prior to sample collection. A detailed physical examination, sport concussion assessment Tool 3 (SCAT3), and past medical history were completed by a sports medicine physician. This included a history of previous head injuries, description of recovery from previous head injuries, neurological conditions, medications, sport participation, and biographical information.

#### Post-SRC Assessments

Athletes were monitored throughout the 2015–2016 sport season. Following a suspected SRC, athletes were assessed by a certified sport medicine physician at the WinSport Medicine Clinic within 72 h of injury and a SCAT3 was administered. Diagnosis of SRC was based on the Consensus statement on concussion in sport: the 4th International Conference on Concussion in Sport held in Zurich, November 2012 criteria (27). Athletes diagnosed with an SRC were asked to provide a 12-h fasting urine sample between 7 and 9 a.m. within a time window of 24 and 72 h post-injury. Athletes were instructed to wipe the urethral opening with an antiseptic alcohol tissue and allow the passage of urine for 3 s before filling the collection cup approximately halfway. All samples were immediately stored at −80°C, batched, and sent to the University of Lethbridge, Lethbridge, Alberta, Canada, for analysis. Symptom evaluation was based on section Result of the SCAT3. The total number of symptoms was evaluated out of 22, and the overall symptom severity was evaluated out of 132 (27). Time to return to play was determined by the treating certified sports medicine physicians based on the return to play guidelines provided by Consensus statement on concussion in sport: the 4th International Conference on Concussion in Sport held in Zurich, November 2012 criteria (27). The number of day to return to play was a reflection of the number of days it took for an athlete to be asymptomatic and to be cleared by the treating physician to start the return to play protocol (27).

### Sample Preparation

In preparation for ^1^H NMR spectroscopy, 400-μl aliquots of urine were added to 200-μl aliquots of phosphate urine buffer in 2-ml centrifuge tubes. The phosphate buffer was prepared as a 4:1 ratio of KH_2_PO_4_ in a 4:1 H_2_O:D_2_O solution to a final concentration of 0.5 M. D_2_O included 0.05% (by weight) trimethylsilyl propanoic acid (TSP) as a chemical shift and concentration reference. To protect the metabolite profile integrity, 0.02% (weight/volume) of sodium azide (NaN_3_) was added to the solution as an antimicrobial agent. The buffer solution was then titrated to pH 7.4 using HCl or NaOH, depending on the initial pH. The tubes containing urine and buffer were centrifuged at 12,000 rpm for 5 min at 4°C. After centrifugation, 550 μl of the supernatant was transferred to a 5-mm NMR tube for NMR analysis. Samples were immediately analyzed using the NMR spectrometer.

### NMR Data Acquisition and Processing

NMR spectra were collected on a 700-MHz Bruker Avance III HD spectrometer (Bruker, ON, Canada). The Bruker 1D NOESY gradient water suppression pulse sequence was used. Each sample was run for 128 scans, and the total acquisition size was 128 k. The spectra were zero filled to 256 k, automatically phased, baseline corrected, and line-broadened to 0.3 Hz. The processed spectra were exported as ascii files to MATLAB version R2015b (The MathWorks, MA, USA) for statistical analysis. Spectra were first binned using dynamic adaptive binning [code available for download at (28)] and then manually adjusted to optimize the number of variables and ensure accuracy. All spectra had the regions corresponding to water and urea removed before normalization to the total area of all spectral bins. The peaks corresponding to acetaminophen derivatives (29) were also removed to account for the fact that the athletes tended to take pain medication following an SRC. The data were then Pareto scaled to increase the influence of weak peaks and deemphasize the influence of larger peaks. All the peaks in each spectrum were referenced to TSP (0.00δ).

### Statistical Analysis

Data visualization to determine the sample structure and the presence of distinct groups was performed using principal component analysis (PCA) and partial least square discriminant analysis (PLS-DA). Ten-fold double cross-validation and 2,000 permutation tests were performed to validate the results of the supervised PLS-DA testing. This followed the recommendations found in literature for validating PLS-DA models (30, 31). Variable importance to the projection (VIP) scores indicate which spectral bins (hereafter referred to as features) contributed the most to the separation observed in the PLS-DA score plot. This score represents a weighted sum of squares of the PLS-DA loadings in each dimension. All tests were performed using the online metabolomics statistics software platform MetaboAnalyst (metaboanalyst.ca) (32–35).

To focus on potential biomarkers, variable importance analysis based on random variable combination (VIAVC) was used as a feature selection tool (36). The VIAVC algorithm combines random permutations of variable inclusion with a 10-fold cross-validation of model. This reveals a best subset of metabolites which have the greatest effect on group differences. VIAVC *p*-values were calculated using a *t*-test and distribution of how many times a metabolite was removed to improve the model during various permutations. Each metabolite in the best subset generated by the algorithm was therefore strongly informative in separating the samples into groups, and synergistic effects between metabolites were revealed. All VIAVC tests were carried out using the MATLAB code provided in the Supplementary Materials of the VIAVC manuscript (36) and MATLAB version R2015b. To further test for significant features between pre-injury and post-SRC, univariate paired *t*-tests were also completed on each spectral bin.

The VIAVC method, VIP scores, and paired *t*-tests each provided a set of features that were considered significantly altered across the comparison groups. The metabolites corresponding to these features were identified using the profiler tool in Chenomx NMR Suite version 8.5 (Chenomx Inc., Edmonton, AB, Canada). Receiver operator characteristic (ROC) curves were also used as they graphically represent the true positive rate vs. false positive rates (37). The accuracy of a classifier was visualized by the area under the ROC curve.

The biological significance of important metabolites was investigated using the free pathway topology analysis tool available through MetaboAnalyst (metaboanalyst.ca) (32). Data were collated as a list of metabolites, and the human pathway library was chosen. The library was built using the detailed Kyoto Encyclopedia of Genes and Genomes (KEGG) pathway diagrams. A list of the most relevant biological pathways involved in conditions of the study was then generated to help draw connections between potential biomarker metabolites and relevant biological processes.

Spearman's correlations compared the normalized concentration of significantly altered metabolites post-SRC to the total number of symptoms reported, the symptom severity score, and the length of return to play. A *p* < 0.05 was considered significant, and metabolite concentrations were normalized with respect to the entire urinary metabolome.

## Results

### Subjects

Forty of the 423 participants sustained a SRC. Of these, 14 were unable to provide two urine samples or did not meet inclusion criteria, resulting in a sample size of 26. Athletes sustaining an SRC but not meeting inclusion criteria (ice hockey participants; mean age 15.15 ± 1.32; 3 females) were comparable to the study population (24 ice hockey participants, 1 luge athletes, 1 taekwondo athlete; mean age 14.72 ± 1.13; 26 male). The most common reason for loss of sample was logistical (athlete absent on day of collection or scheduling conflicts); however, there may be an intrinsic difference between these two populations contributing to selection bias. Patient characteristics including age, sex, medical history, medication (prescribed and over-the-counter medication), supplements, and sport participation are in Table 1. The length of loss of consciousness, length of posttraumatic amnesia, pre and post number of symptoms, symptom severity [Symptom evaluation section of the Sport-concussion Assessment Tool−3 (SCAT3)], and length of return to sport are in Table 2 for all participants. There were no other injuries at the time of SRC.

**Table 1:** Patient characteristics.

**Subject number**	**Age**	**Past medical history**	**Medications**	**Number of previous concussions**	**Sport participation**
1	15	-	-	1	Ice hockey
2	16	Asthma	Ventolin, protein powder	0	Ice hockey
3	14	-	Vitamin D, coenzyme Q10, vitamin C	0	Ice hockey
4	16	Asthma	Ventolin, protein powder	0	Ice hockey
5	16	Acne	Biosteel protein, minocycline	0	Ice hockey
6	16	-	-	2	Ice hockey
7	16	Depression	Protein powder, creatine	1	Ice hockey
8	16	-	Mesavant	0	Ice hockey
9	15	Migraine headaches	-	1	Ice hockey
10	14	Migraine headaches	-	3	Ice hockey
11	15	Generalized anxiety disorder, asthma	Ventolin	1	Ice hockey
12	13	-	-	0	Ice hockey
13	13	-	-	0	Ice hockey
14	15	-	Naturopathic growth hormone	0	Ice hockey
15	13	-	-	0	Ice hockey
16	13	-	Vegan protein	0	Ice hockey
17	16	Acne, previous whiplash injury of the neck	Acutane	0	Ice hockey
18	14	-	-	0	Luge
19	15	-	-	1	Ice Hockey
20	14	-	-	1	Ice hockey
21	12	Migraine headaches	-	0	Taekwondo
22	14	-	Vitamin D, vitamin B, protein powder	0	Ice Hockey
23	15	-	Protein powder	1	Ice hockey
24	16	Acne, migraine headaches	Minocycline	0	Ice hockey
25	15	-	Protein powder, vitamin C, vitamin D, creatine	0	Ice hockey
26	13	-	Cod liver oil, vitamin D	1	Ice hockey

**Table 2:** Symptom scores, length of return to sport, length of loss of consciousness, length of post-traumatic Amnesia.

**Subject number**	**Number of symptoms at time of injury**	**Symptom severity score at the time of injury**	**Number of symptoms when cleared to Sport**	**Symptom severity score when cleared to Sport**	**Length of loss of consciousness (seconds)**	**Length of post traumatic amnesia (minutes)**	**Length of return to sport (days)**
1	0	0	0	0	0	1	10
2	13	22	0	0	0	0	9
3	0	0	0	0	0	0	2
4	13	22	0	0	0	0	9
5	20	65	0	0	0	0	17
6	2	4	2	4	0	0	3
7	11	16	2	2	0	0	11
8	15	35	3	3	0	0	23
9	8	16	0	0	0	0	8
10	13	44	1	4	0	0	78
11	3	5	0	0	0	0	17
12	0	0	0	0	0	0	12
13	0	0	0	0	0	0	13
14	18	47	0	0	0	0	16
15	15	28	1	2	0	0	10
16	7	14	0	0	0	0	38
17	1	1	0	0	0	0	10
18	10	10	0	0	180	3	30
19	16	47	2	2	0	0	36
20	6	9	0	0	0	0	18
21	12	18	1	1	0	0	42
22	9	21	0	0	0	0	9
23	4	8	1	2	0	0	19
24	20	22	0	0	0	1	18
25	2	3	2	2	10	20	10
26	11	22	0	0	0	0	17

### Metabolomic Outcomes

Initially, exploratory analysis using both supervised and unsupervised models of all data was completed. The unsupervised PCA was used to cluster the raw data and highlight any separation between groups. The PCA scores plot revealed only a slight group difference (Supplementary Figure 1). Next, supervised PLS-DA clustering analysis was performed, revealing a distinct separation between the pre-injury and post-SRC groups (Supplementary Figure 2). However, the data did not pass permutation testing. Therefore, VIAVC was used to remove unimportant or interfering features from the data. VIAVC left a subset of 18 features that have a strong effect on the differences between pre-injury and post-SRC. The VIAVC *p*-value represents a measurement of whether the inclusion of a feature in 1,000 randomly chosen subsets of features improved or reduced the overall class separation of the model. Thus, VIAVC is able to determine synergistic effects across features.

**Figure 1:**
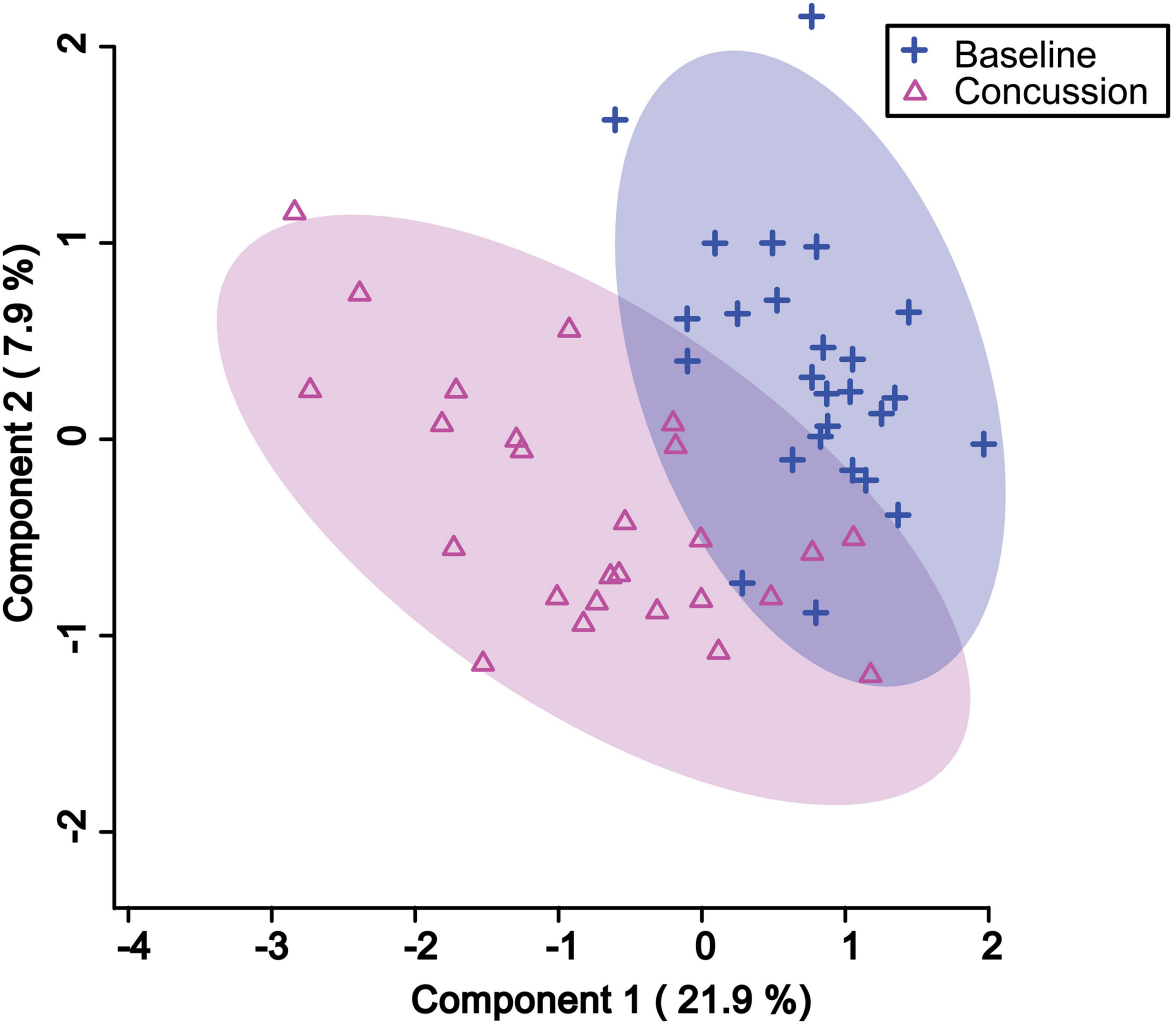
Partial least square discriminant analysis (PLS-DA) 2D score plot showing the separation between pre-injury and post-SRC urine samples based on the VIAVC best subset of features corresponding to the metabolites provided in Table 3. The percentages shown along the axis indicate the amount of variance in the data set given by each component, and the shaded ellipses designate the 95% confidence interval of each group.

**Figure 2:**
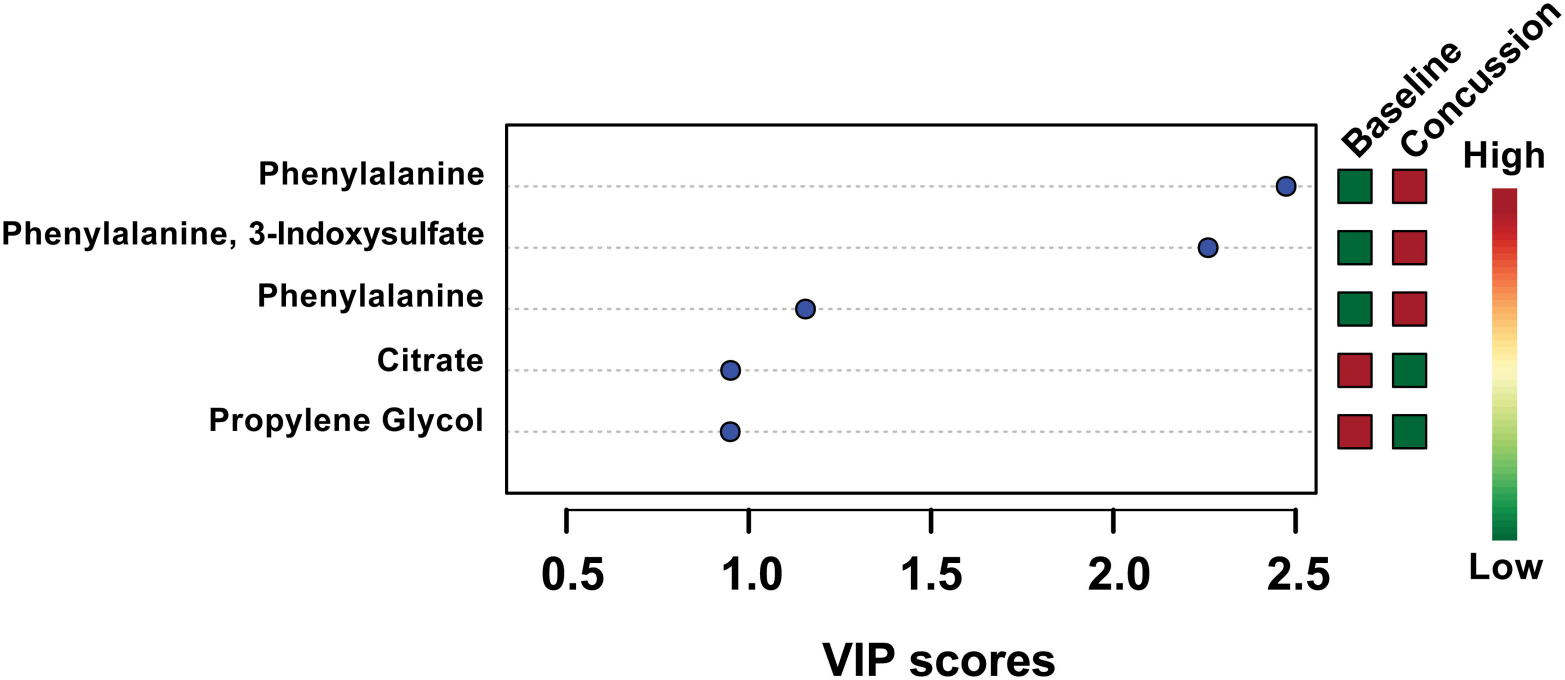
Variable importance in the projection (VIP) scores for the top five metabolites used in the PLS-DA model. The higher the VIP score, the more the metabolite(s) contributed to the separation observed between groups in the PLS-DA model shown in Figure 1. The colored boxes on the right side indicate whether the metabolite was upregulated or downregulated in the post-SRC sample with respect to the pre-injury sample.

PLS-DA analysis was completed again, this time focusing only on the 18 features identified by VIAVC. A clear separation was found between groups (Figure 1) with Components 1 and 2 accounting for 21.9 and 7.9% of the variance in the data, respectively. The model passed the permutation testing using 2,000 permutations (*p* = 0.0005), confirming that the separation is real and not due to chance. This model also passed the 10-fold cross-validation testing. Figure 2 provides a VIP score plot illustrating the top five features and the metabolites corresponding to each feature. The heat map (Figure 2) indicates the directionality of the changes, i.e., upregulation or downregulation by SRC. Figure 2 also indicates that phenylalanine and 3-indoxysulfate are the top two metabolites leading to the observed separation and have been upregulated following SRC. Citrate and propylene glycol are the next most important metabolites to the observed group separation and have been downregulated following an SRC.

**Figure 3:**
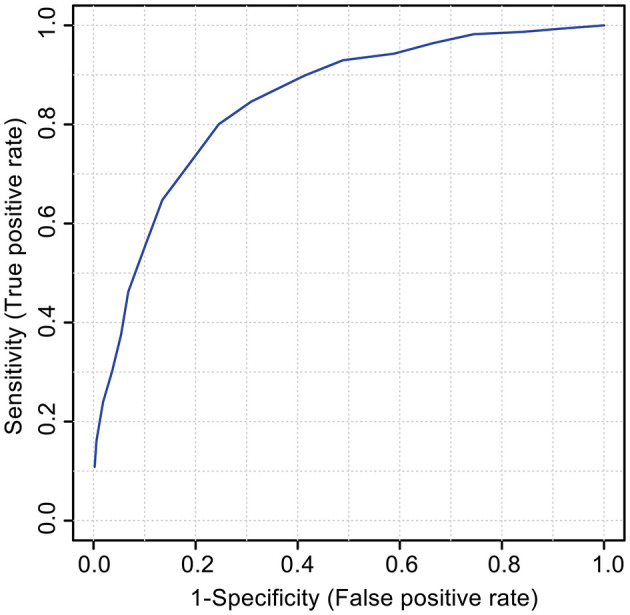
The receiver operator characteristic (ROC) curve for the VIAVC best subset of features corresponding to the metabolites provided in Table 3. The model graphs the true positive rate on the y-axis vs. the false-positive rate on the x-axis. The corresponding area under the curve (AUC) was 0.887 with a 95% confidence interval of 0.731–0.997, and the model had a predictive accuracy of 81.6%.

An ROC was constructed to test if the 18 features identified as the VIAVC best subset can be used to accurately predict whether a sample belongs to the pre-injury or post-SRC group. The ROC model had a corresponding area under the curve (AUC) of 0.887 with a 95% confidence range of 0.731–0.997 and a predictive accuracy of 81.6% (Figure 3).

**Table 3:** List of metabolites corresponding to the 30 features that were significantly changed following SRC based on either the VIAVC best subset analysis or a paired t-test.

**Metabolite**	**VIAVC *P*-value**	**PLS-DA VIP Score**	***T*-Test *P*-value**	**Regulation**
2-Hydroxybutyrate	5.42E-45	0.41803	Not Sig.	Down
3,4-Dihydroxybenzeneacetate.1/Carnitine.1	4.86E-19	0.14659	Not Sig.	Up
4-Hydroxybenzoate	3.98E-85	0.58827	Not Sig.	Up
Caffeine	3.29E-52	0.35628	Not Sig.	Down
Carnitine.2	1.56E-38	0.54019	Not Sig.	Up
Homocitrulline	2.15E-77	0.44871	Not Sig.	Down
Methionine/Acetylcarnitine.1	2.59E-50	0.4312	Not Sig.	Up
3-Methyl-2-Oxovalerate	8.18E-26	0.41031	Not Sig.	Up
Phosphorylcholine/Choline/Acetylcarnitine.2	1.52E-128	0.55238	Not Sig.	Up
Propylene Glycol	4.50E-77	0.94917	Not Sig.	Down
Taurine/3,4-Dihydroxybenzeneacetate.2/Carnitine.3	1.40E-41	0.37166	Not Sig.	Up
*1-Methylhistadine/3-Methylhistadine	3.69E-50	0.91339	0.03871	Down
*Citrate	9.08E-100	0.95022	0.032117	Down
*Lactose	8.24E-43	0.78734	0.043613	Up
*Phenylalanine.1	1.60E-43	1.1556	0.00719	Up
*Phenylalanine.2	1.71E-42	2.4739	0.000322	Up
*Phenylalanine.3/3-Indoxylsulfate.1	2.30E-72	2.26	0.002267	Up
*Sucrose	2.22E-42	0.62691	0.037745	Down
3-Methyladipate/Isobutyrate	Not Sig.	N/A	0.04133	Down
3-Hydroxyisovalerate	Not Sig.	N/A	0.020594	Down
3-Indoxylsulfate.2	Not Sig.	N/A	0.006534	Up
5-Aminolevulinate	Not Sig.	N/A	0.019592	Up
Anserine.1/Tyrosine	Not Sig.	N/A	0.026885	Up
Anserine.2	Not Sig.	N/A	0.037285	Up
Carnosine/Anserine.3	Not Sig.	N/A	0.024957	Up
Isoleucine/Leucine	Not Sig.	N/A	0.02161	Up
Phenylalanine.4	Not Sig.	N/A	0.002615	Up
Phenylalanine.5	Not Sig.	N/A	0.005616	Up
Phenylalanine.6	Not Sig.	N/A	0.033934	Up
Threonate/Cysteine	Not Sig.	N/A	0.011865	Down

*Each feature's VIAVC p-value, VIP score from the PLS-DA analysis, paired t-test p-value, and regulation are shown. Any metabolites that have a number following their name (for example, Phenylalanine.1 and Phenylalanine.2) correspond to different spectral peaks of the same metabolite. Asterisks indicate that this feature was common to both VIAVC and paired t-test. Entries labeled with Not Sig. correspond to metabolites that were not found to be significant by the corresponding test. Entries labeled N/A indicate that a VIP score was not calculated on those metabolites, as they were not included in the PLS-DA model shown in Figure 2*.

A paired *t*-test was also completed to compare pre-injury and post-SRC samples of each athlete. This test identified 19 features as significant (*p* <0.05), with seven of these features being common to the VIAVC results. Table 3 provides a list of the 31 metabolites identified as significant by VIAVC or a paired *t*-test or both tests.

**Figure 4:**
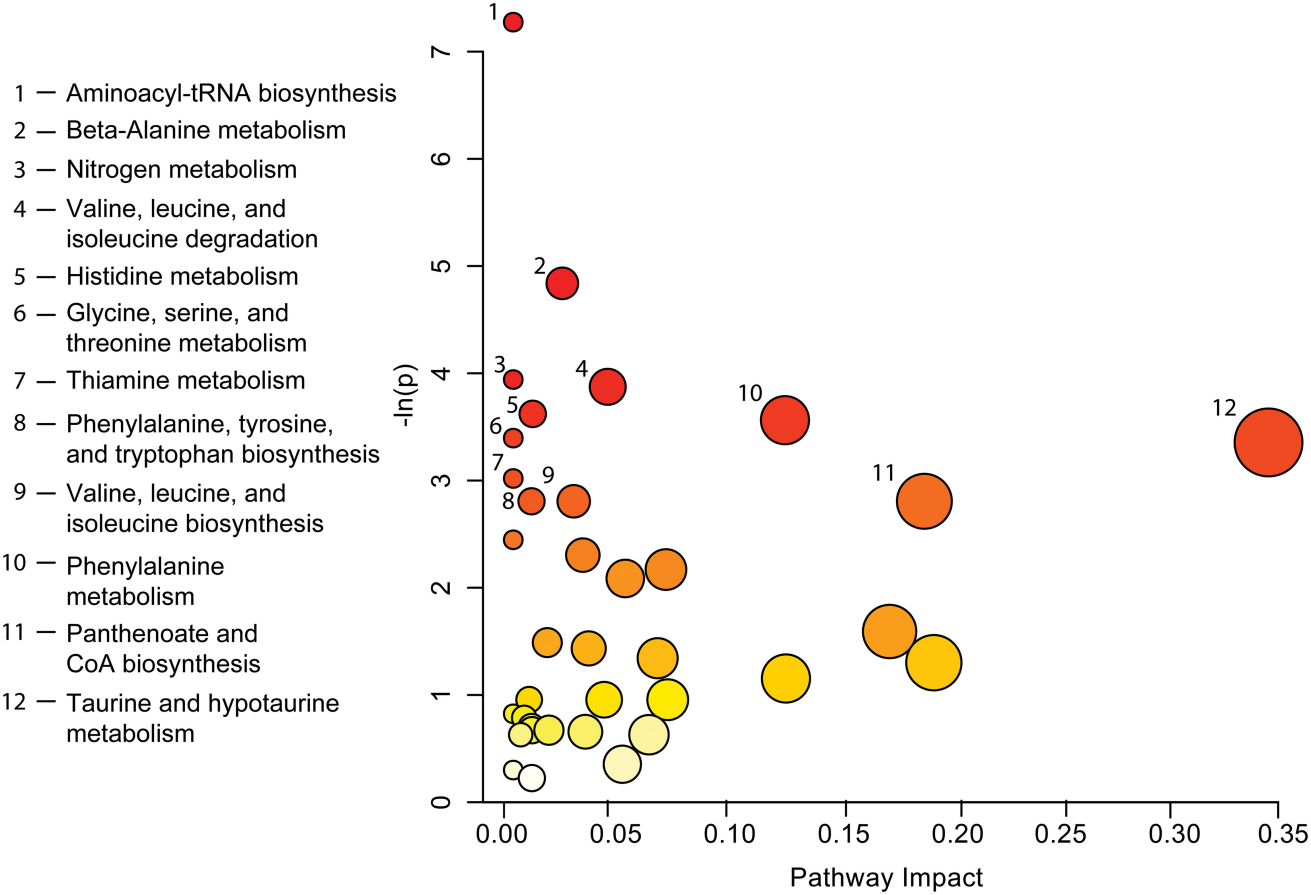
Pathway topology analysis completed by entering a list of significant metabolites found by both VIAVC and paired *t*-test (Table 3) into the web-based tool MetPA. Each circle on the chart indicates a specific metabolic pathway or biological function as labeled to the left of the chart. The *x*-axis indicates the pathway impact scores, which represents the magnitude of impact by significant metabolites, as shown by the size of each circle. The *y*-axis corresponds to the *p*-values, given as -ln(p), with red circles indicating a lower *p*-value and yellow a higher *p*-value. Only pathways with *p* ≤ 0.06 have been labeled.

A complete list of metabolites identified by both methods (see Table 3) was used to carry out pathway topology analysis (Figure 4) and highlights which metabolic processes may be most affected following a concussion. Aminoacyl-tRNA biosynthesis (*p* <0.001) and beta-alanine metabolism (*p* <0.01) were the two pathways with the most significant change. Taurine and hypotaurine metabolism, panthenoate and CoA biosynthesis, and phenylalanine metabolism (*p* <0.005 and pathway impact of 0.11906) show the highest pathway impacts.

### Clinical Correlations

There was a significant positive correlation between the post-SRC normalized concentration of 2-hydroxybutyrate and the length of return to play (ρ = 0.482, *p* = 0.02). Further, there was a significant positive correlation between the total number of symptoms and the post-SRC normalized concentration of lactose (ρ = 0.422, *p* = 0.036).

## Discussion

This is the first study to evaluate alterations in urine metabolite profiles in training male athletes pre-injury compared to post-SRC using a within-subject design. Several metabolites involved in key biological pathways critical to primary and secondary central nervous system injury and recovery processes were altered following SRC. The present data provide a panel of 18 urinary metabolites that as a group have the potential to provide a biomarker signature to aid in the diagnosis of SRC. Finally, exploratory analysis revealed that three specific metabolites significantly correlated with increased symptom burden and prolonged return to sport.

Although the use of metabolomics to investigate injuries to the brain is a relatively novel approach, liquid- or gas-phase chromatography combined with mass spectroscopy has shown promising results in previous studies of psychiatric and neurological disorders (38–41). As opposed to other techniques, ^1^H NMR spectroscopy of urine allows for high-throughput biofluid analysis with a broad, untargeted approach to biomarker discovery. Previous studies exploring metabolomics in SRC have focused on whole blood or serum; urine alternatively represents a clinically accessible, painless, noninvasive medium for metabolic studies. Urine is comprised of the biological by-products produced throughout the body, including the brain (20).

Many metabolites that are present and identifiable by NMR in serum and CSF are also present and identifiable by NMR in urine. In fact, 91.8% (45/49) of the metabolites that are identifiable by NMR in serum and 81.0% (43/53) of the metabolites that are identifiable by NMR in CSF are present and identifiable by NMR in urine (25, 42, 43). Therefore, many of the metabolomic changes that are observed in blood and cerebrospinal fluid should also be detectable in urine. Furthermore, 209 metabolites are identifiable by NMR in urine, meaning that urine not only contains most of the metabolomic information provided by blood and CSF but also provides more additional information about the metabolome compared to the other two biofluids (25). For this study, of the 31 metabolites identified (see Table 3), 18 were unique to urine and 13 could be found in both urine and/or CSF and serum. Therefore, analysis of urine metabolites holds promise as a tool to better understand the pathophysiology changes that can occur following SRC as it is an easily accessible biofluid medium, uses a broad untargeted approach providing a wide breadth of metabolites to study, and has been previously explored in other neurological conditions.

### Metabolites Significantly Altered Following SRC

Arguably, an effective diagnostic tool for SRC will be based on a group of several markers to generate a biomarker signature in conjunction with a clinical assessment (44). The ROC analysis shown in Figure 3 illustrates that the combination of the 18 metabolites identified in Table 3 serves as a classifier of SRC in urine. The particularly relevant metabolic pathways and their role in SRC are discussed below.

Multiple metabolites were significantly downregulated in athletes following SRC, notably 2-hydroxybutyrate, citrate, propylene glycol, valine, 1-methylhistadine, and 3-methyhistadine. Previous studies in concussion (38), mild–severe traumatic brain injury (TBI) (38, 41, 45), and other neurological disorders (20) have also found these metabolites to be significantly altered. Importantly, a study by Dash et al. (38) found serum 2-hydroxybutyrate to be significantly decreased post-TBI compared to healthy controls and was positively correlated with the severity of head injury. Similarly, a significant reduction in plasma citrate has been found in patients with moderate–severe TBI (41), as well as in stroke (20). Furthermore, similar to our study Daley et al. (10) reported that plasma propylene glycol contributed to the 10 components that explained a significant variance between concussed and non-concussed male hockey players. In contrast, Glenn et al. (45) found a significant increase, not decrease, in propylene glycol in patients with severe TBI. Of the metabolites downregulated in this study, 2-hydroxybutyrate and citrate have implication in oxidative stress pathways. Urinary excretion of 2-hydroxybutyrate is known to reflect shifts in the rate of glutathione synthesis, reflecting alterations in oxidative stress (46). As well, previous work has shown that citrate chelation is involved in peroxidation rates contributing to an increase in oxidative stress (47–49). Oxidative stress has been explored as one of the many cascades of pathophysiological changes that can occur following concussion, suggesting the downregulation of these metabolites which may be markers of this process (50).

There were many urine metabolites that were significantly upregulated in athletes following SRC, specifically phenylalanine, 3-indoxysulfate, phosphorylcholine, methionone, tyrosine, anserine, carosine, carnitine, acetylcarnitine, 3,4-dihydroxybenzeneacetic acid, leucine, isoleucine, and valine. Of these, phenylalanine and 3-indoxysulfate were the most significantly altered. Previous studies exploring phenylalanine following brain injury are varied. Similar to our study, Bahado-Singh et al. (51) found a significant upregulation of phenylalanine in a concussed mouse model. In contrast, Yi et al. (41) observed a significant decrease in phenylalanine in patients following moderate–severe TBI and concurrent cognitive impairment. Furthermore, Viant et al. (52) and Dash et al. (38) found changes in phosphorylcholine in brain tissue in a fluid percussion rat model of head injury and an upregulation in plasma methionine in patients with TBI. Similar to our study, alterations in anserine, carnosine, carnitine, acetylcarnitine, tyrosine, methionine, and 3,4-dihydroxybenzeneacetic acid have been implicated as protected or upregulated in a variety of disorders including Alzheimer's disease (19, 53–57), ischemic stroke (58–60), cardiovascular ischemic damage (53), chronic fatigue syndrome (61), major depressive disorder (62), and inflammatory diseases (53). This suggests that these metabolites may be increased following SRC as a potential protective mechanism following injury. Finally, leucine and isoleucine, branched-chain amino acids (BCAA), were upregulated post-SRC. These BCAA are particularly involved in stress, energy, and muscle metabolism. In contrast to our findings, Vuille-Dit-Bille et al. (63) found that plasma BCAA were significantly decreased in patients with TBI. Interestingly, BCAA are transported across the blood–brain barrier by the same, competitive mechanism as aromatic amino acids (AAA) such as phenylalanine and tyrosine (64). As noted above, these AAA were also significantly altered following SRC and are important to the synthesis and release of the catecholamine neurotransmitters DA and NA.

### Urine Metabolites and Clinical Correlations

Metabolites that were significantly altered post-SRC were correlated with clinical outcomes of symptom burden and length of return to play. A positive correlation was found between 2-hydroxyisobutyrate and length of return to play. Similarly, Dash et al. found that 2-serum hydroxyisobutyrate was positively correlated with severity of TBI. This suggests that 2-hydroxybutyrate is a metabolite of interest when determining recovery and prognosis following head injuries of all severities and warrants further study in this domain. This study also found a significant positive correlation between lactose and the total number of symptoms post-SRC. Similarly, Vitek et al. (65) found urine lactulose to be elevated in patients with TBI compared to healthy controls and patients with extra-cerebral injuries. As well, they found that the more severe the TBI, the higher the urine lactulose, and hypothesized that an increase in lactulose may reflect increased catabolism of brain gangliosides following injury.

### Strength and Limitations

There were multiple strengths and some limitations apparent in this study. The within-subject design was unique as it compared samples in non-injured training athletes during their athletic season to acute post-SRC in the *same person*. This approach reduces between-sample bias and physiological variations due to age, sex, and individual lifestyle. Furthermore, the collection of morning samples reduces the impact of diurnal hormonal variation and exercise. There were multiple limitations to the study including a small sample size, with no female participants, collection of samples at only two time points (pre- and post-acute injury), and a lack of a healthy control group or comparison to other injury group such as an MSK-injured group. Future studies should include a collection of multiple samples both pre- and post-injury in the same athlete compared to healthy controls and musculoskeletal injury athletes with a much larger sample size in both males and females. The female athlete menstrual cycle should also be taken into consideration. As well, dietary contributions were unknown and not accounted for. Further, due to the small sample size it is difficult to determine if our results were influenced by pre-morbid medical factors such as a history of migraine headaches or mental health difficulties. Finally, urine metabolite analysis is novel, providing an easily accessible, painless noninvasive biofluid that is relatively far downstream from the site of injury, allowing for an increasing number of variables to alter the outcome.

Future studies would benefit from a larger sample size and collection of multiple post-SRC samples throughout recovery of both sexes compared to both a healthy control group and a non-SRC-injured group such as a musculoskeletal injury. This study would also control for dietary contributions, fluid intake, and past medical history.

## Conclusion

In conclusion, 18 urinary features were identified by VIAVC, clearly separating between pre-injury training athletes and post-SRC samples in the same individual using PLS-DA clustering analysis. The model passes permutation and cross-validation testing, and an ROC curve shows that the best subset was a good classifier of SRC with an AUC of 0.887. Furthermore, this study provides a significant correlation between measures of symptom burden and return to play and urinary metabolite concentrations, thus providing support for the potential use of urinary metabolomic biomarkers in prognosticating outcomes following SRC. Discovery of the urinary metabolomic biomarker signature for SRC has the potential to provide an objective, accurate, and cost-effective method to aid in the diagnosis and monitoring of concussions at the clinical level. The strengths of our study include the stringent timing and collection of the samples, the within-person comparison, the ease at which urine can be collected compared to blood, and the novel analytical approach.

## Data Availability Statement

The original contributions presented in the study are included in the article/Supplementary Material, further inquiries can be directed to the corresponding author/s.

## Ethics Statement

The studies involving human participants were reviewed and approved by University of Calgary Conjoint Ethics Board. Written informed consent to participate in this study was provided by the participants' legal guardian/next of kin.

## Author Contributions

GM, CD, TM, SD, and BB conceptualized and designed the study and recruited the participants. NB, ZW, and EP assisted with developing the methods and collecting the experimental data. PS and TM carried out the correlation analysis of metabolomic data to the clinical measures. ZW, CS, PS, TM, SD, GM, and CD contributed to writing the main manuscript. All authors contributed to the article and approved the submitted version.

## Conflict of Interest

The authors declare that the research was conducted in the absence of any commercial or financial relationships that could be construed as a potential conflict of interest.

## Publisher's Note

All claims expressed in this article are solely those of the authors and do not necessarily represent those of their affiliated organizations, or those of the publisher, the editors and the reviewers. Any product that may be evaluated in this article, or claim that may be made by its manufacturer, is not guaranteed or endorsed by the publisher.

## Supplementary Material

Supplementary Figure 1 **Principle component analysis (PCA) 2D scores plot showing only minimal separation between baseline and post-SRC urine samples. The unsupervised PCA test was performed using the full data set, without utilizing any feature selection techniques. The percentages shown along the axis indicate the amount of variance in the data set given by each component and the shaded ellipses designate the 95% confidence interval of each group**.

Supplementary Figure 2 **Partial least squares discriminant analysis (PLS-DA) 2D scores plot showing separation between baseline and post-SRC urine samples. The supervised PLS-DA test was performed using the full data set, without utilizing any feature selection techniques. The model did not pass permutation or cross validation tests, obscuring a causative interpretation of the data. The percentages shown along the axis indicate the amount of variance in the data set given by each component and the shaded ellipses designate the 95% confidence interval of each group**.
