# Supplementary figures and images for: Alterations in Urine Metabolomics Following Sport-Related Concussion: A 1H NMR-Based Analysis
Source: Front Neurol. 2021 Aug 19;12:645829. doi: 10.3389/fneur.2021.645829 (PMC8416667; doi:10.3389/fneur.2021.645829)

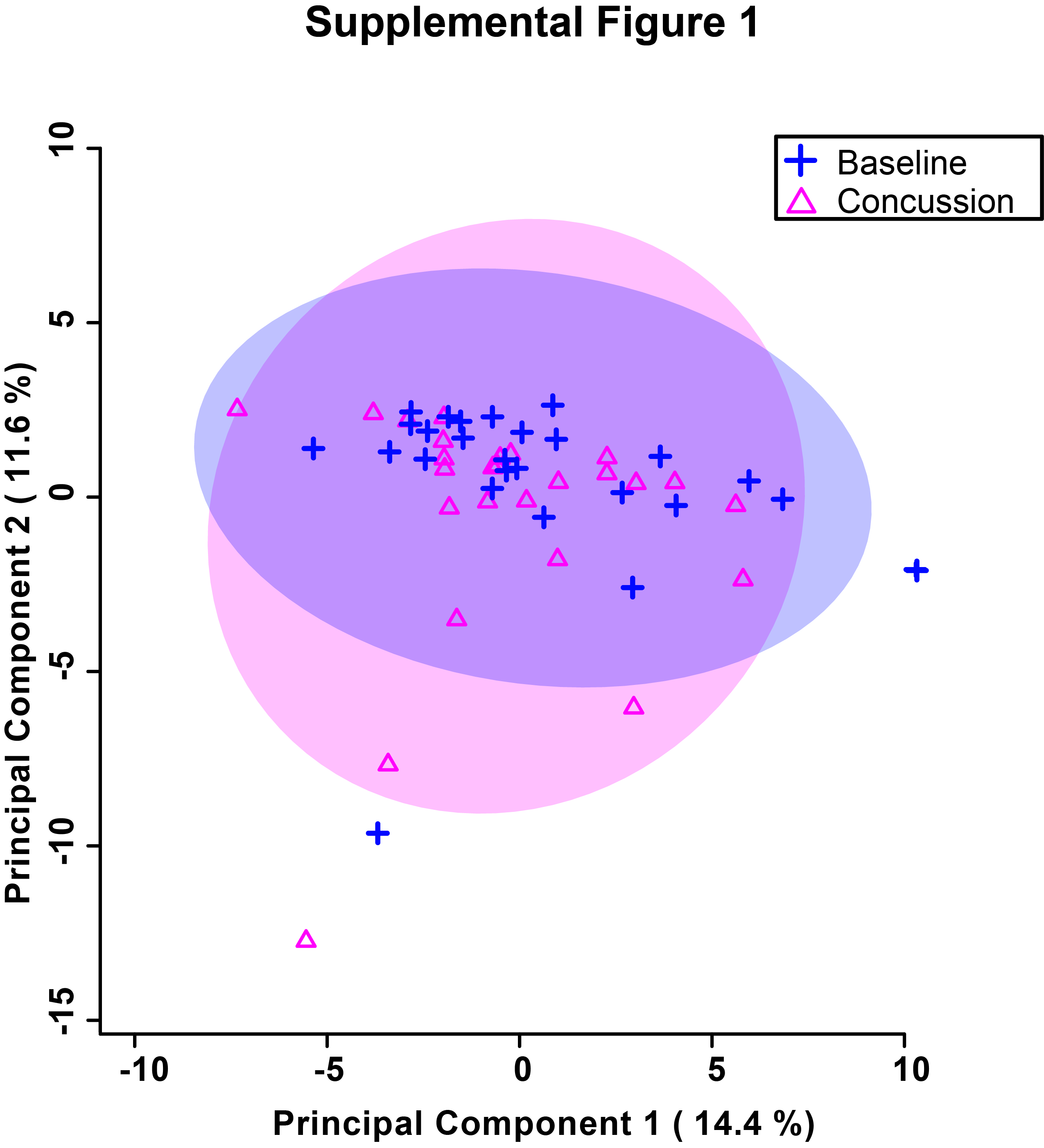

Supplement: Supplementary Figure 1 — Principle component analysis (PCA) 2D scores plot showing only minimal separation between baseline and post-SRC urine samples. The unsupervised PCA test was performed using the full data set, without utilizing any feature selection techniques. The percentages shown along the axis indicate the amount of variance in the data set given by each component and the shaded ellipses designate the 95% confidence interval of each group. [file Image_1.png]

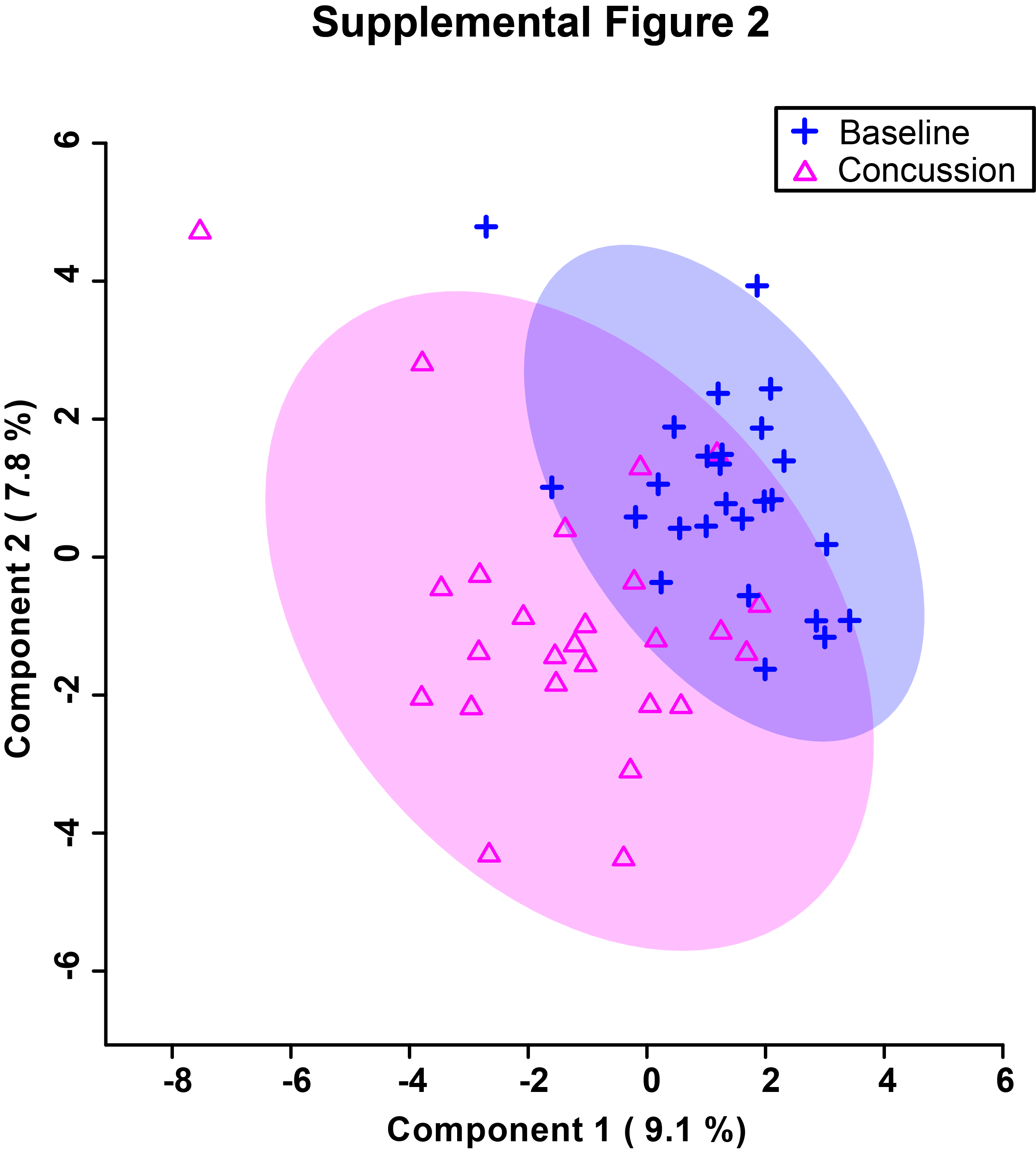

Supplement: Supplementary Figure 2 — Partial least squares discriminant analysis (PLS-DA) 2D scores plot showing separation between baseline and post-SRC urine samples. The supervised PLS-DA test was performed using the full data set, without utilizing any feature selection techniques. The model did not pass permutation or cross validation tests, obscuring a causative interpretation of the data. The percentages shown along the axis indicate the amount of variance in the data set given by each component and the shaded ellipses designate the 95% confidence interval of each group. [file Image_2.png]
